# Supplementary material for: Association between Physical Activity and Phase Angle Obtained via Bioelectrical Impedance Analysis in South Korean Adults Stratified by Sex
Source: Nutrients. 2024 Jul 4;16(13):2136. doi: 10.3390/nu16132136 (PMC11242964; doi:10.3390/nu16132136)
Supplement: Supplementary file 1 [file nutrients-16-02136-s001.zip › Supplementary Table S1.pdf]

Supplementary Table S1. Results of subgroup analysis stratified by independent variables.

Variables	Male					Female				
	Inactive	Insufficiently active		Sufficiently active		Inactive	Insufficiently active		Sufficiently active	
		Above average Phase Angle[a]		Above average Phase Angle[a]			Above average Phase Angle[a]		Above average Phase Angle[a]	
	aOR[b]	aOR[b]	95% CI	aOR[b]	95% CI	aOR[b]	aOR[b]	95% CI	aOR[b]	95% CI
**Age**										
19~28	1.000	2.188	0.624 - 7.666	1.594	0.633 - 4.012	1.000	2.172	0.776 - 6.078	2.809	1.123 - 7.027
29~39	1.000	4.862	1.195 - 19.786	6.136	2.030 - 18.546	1.000	1.460	0.664 - 3.214	1.814	0.938 - 3.508
40~49	1.000	1.657	0.638 - 4.300	3.983	1.749 - 9.071	1.000	0.741	0.381 - 1.441	0.820	0.450 - 1.494
50~59	1.000	0.832	0.350 - 1.975	1.031	0.495 - 2.149	1.000	1.359	0.675 - 2.736	1.300	0.660 - 2.561
60~69	1.000	1.706	0.812 - 3.583	1.789	0.879 - 3.642	1.000	0.917	0.405 - 2.072	1.843	1.066 - 3.187
70~	1.000	2.923	0.849 - 10.070	1.084	0.330 - 3.564	1.000	0.546	0.128 - 2.323	0.627	0.144 - 2.719
**Body Mass Index[c]**										
Underweight	1.000	N.A	N.A - N.A	N.A	N.A - N.A	1.000	0.664	0.144 - 3.061	1.336	0.470 - 3.795
Normal weight	1.000	1.431	0.732 - 2.795	3.583	2.106 - 6.096	1.000	1.361	0.892 - 2.077	1.396	0.947 - 2.059
Overweight	1.000	2.622	1.076 - 6.388	2.471	1.124 - 5.433	1.000	0.778	0.382 - 1.585	1.793	0.960 - 3.346
Obese	1.000	1.598	0.910 - 2.804	1.463	0.825 - 2.594	1.000	0.865	0.427 - 1.752	0.908	0.530 - 1.554
**Education level**										
Lower than middle school	1.000	1.854	0.577 - 5.953	2.389	1.027 - 5.559	1.000	1.677	0.713 - 3.944	1.057	0.570 - 1.962
High school	1.000	1.538	0.874 - 2.708	1.872	1.090 - 3.216	1.000	1.012	0.579 - 1.770	1.490	0.907 - 2.447
College or above	1.000	1.504	0.846 - 2.674	1.952	1.165 - 3.270	1.000	1.089	0.710 - 1.668	1.328	0.936 - 1.884
**Alcohol status[d]**										
Non-drinker	1.000	1.617	0.804 - 3.253	3.282	1.756 - 6.135	1.000	1.071	0.696 - 1.648	1.700	1.138 - 2.539
Social drinker	1.000	1.830	0.893 - 3.752	1.431	0.784 - 2.612	1.000	1.009	0.524 - 1.944	1.214	0.788 - 1.871
Current drinker	1.000	0.992	0.537 - 1.832	1.766	1.033 - 3.020	1.000	1.042	0.386 - 2.815	0.716	0.329 - 1.558
**Smoking status**										
Ever-smoker	1.000	1.767	0.898 - 3.475	3.121	1.614 - 6.034	1.000	1.688	0.054 - 52.428	0.568	0.167 - 1.935
Non-smoker	1.000	1.392	0.883 - 2.194	1.664	1.116 - 2.481	1.000	1.062	0.760 - 1.482	1.359	1.037 - 1.782
**Region of residence**										
Metropolitan	1.000	1.843	1.049 - 3.240	2.471	1.494 - 4.089	1.000	1.074	0.629 - 1.834	1.398	0.959 - 2.039
Urban	1.000	1.371	0.723 - 2.598	1.725	0.905 - 3.285	1.000	0.787	0.496 - 1.249	1.080	0.699 - 1.669
Rural	1.000	2.093	0.907 - 4.826	3.286	1.021 - 10.575	1.000	2.698	1.361 - 5.349	1.596	0.755 - 3.374
**Marital status**										
Married	1.000	1.478	0.918 - 2.380	1.871	1.193 - 2.932	1.000	0.943	0.646 - 1.376	1.332	0.932 - 1.901
Single	1.000	1.677	0.830 - 3.389	1.943	1.067 - 3.538	1.000	1.407	0.774 - 2.558	1.419	0.880 - 2.290
**Income level[e]**										
Low	1.000	0.905	0.343 - 2.389	1.871	0.905 - 3.865	1.000	0.695	0.326 - 1.481	0.746	0.441 - 1.259
Middle low	1.000	2.586	1.084 - 6.172	1.875	0.923 - 3.810	1.000	1.058	0.549 - 2.041	1.388	0.809 - 2.381
Middle high	1.000	1.925	0.927 - 3.997	2.625	1.363 - 5.057	1.000	0.822	0.429 - 1.576	1.306	0.772 - 2.208

High	1.000	1.608	0.795	-	3.251	1.899	1.161	-	3.108	1.000	1.461	0.751	-	2.841	1.810	1.038	-	3.157
**Employment status**																		
Employed	1.000	1.542	1.007	-	2.361	2.065	1.389	-	3.071	1.000	1.055	0.699	-	1.593	1.243	0.877	-	1.763
Unemployed	1.000	1.849	0.610	-	5.608	1.277	0.570	-	2.863	1.000	1.093	0.667	-	1.790	1.428	0.928	-	2.197
**Sleep duration**																		
<6 hours	1.000	2.589	0.640	-	10.480	7.393	2.550	-	21.434	1.000	0.543	0.180	-	1.640	1.639	0.775	-	3.466
≥6 hours and <8 hours	1.000	1.597	1.007	-	2.530	1.878	1.233	-	2.859	1.000	1.077	0.744	-	1.559	1.142	0.809	-	1.610
≥8 hours and <10 hours	1.000	1.248	0.492	-	3.165	1.899	1.063	-	3.393	1.000	1.197	0.672	-	2.135	1.641	0.889	-	3.031
≥10 hours	1.000	N.A	N.A	-	N.A	3.072	0.186	-	50.648	1.000	N.A	N.A	-	N.A	N.A	N.A	-	N.A
**Diabetes**																		
No	1.000	1.648	1.087	-	2.497	2.124	1.449	-	3.113	1.000	1.080	0.770	-	1.515	1.432	1.090	-	1.882
Yes	1.000	1.160	0.441	-	3.050	1.175	0.465	-	2.968	1.000	1.442	0.297	-	6.996	0.619	0.272	-	1.412
**High Blood pressure**																		
No	1.000	1.818	1.133	-	2.920	2.585	1.693	-	3.947	1.000	1.099	0.757	-	1.595	1.330	0.981	-	1.805
Yes	1.000	1.216	0.600	-	2.467	0.933	0.517	-	1.682	1.000	1.078	0.527	-	2.208	1.269	0.695	-	2.318
**Asthma**																		
No	1.000	1.479	0.998	-	2.192	2.001	1.407	-	2.846	1.000	1.056	0.762	-	1.465	1.335	1.022	-	1.744
Yes	1.000	N.A	N.A	-	N.A	N.A	N.A	-	N.A	1.000	N.A	N.A	-	N.A	N.A	N.A	-	N.A
**Kidney Disease**																		
No	1.000	1.482	1.011	-	2.172	1.953	1.371	-	2.782	1.000	1.073	0.778	-	1.480	1.320	1.003	-	1.737
Yes	1.000	N.A	N.A	-	N.A	N.A	N.A	-	N.A	1.000	N.A	N.A	-	N.A	N.A	N.A	-	N.A

aOR, adjusted odds ratio; CI, confidence interval; N.A., not applicable.

N.A. is due to small sample size.

[a]Average phase angle: 5.77° for males and 4.88° for females

[b]Adjusted for the amount of physical activity, age, body mass index, educational level, alcohol status, smoking status, region of residence, marital status, income level, employment status, sleep duration, presence of diabetes, high blood pressure, asthma, and kidney disease.

[c]'Underweight': <18.5 kg/m^2, 'normal weight': between 18.5 kg/m^2 and 23.0 kg/m^2, 'overweight': between 23.0 kg/m^2 and 25.0 kg/m^2, and 'obese': >25.0 kg/m^2

[d]'Non-drinker': does not drink at all, 'social drinker': drinks once or twice every month, and 'current drinker': drinks every week

[e]Categorised into 'low', 'middle low', 'middle high', and 'high' if the income percentile of the participant was <25th percentile, >25th percentile and <50th percentile, >50th percentile and <75th percentile, and >75th percentile, respectively.
