# Supplementary material for: Association between Physical Activity and Phase Angle Obtained via Bioelectrical Impedance Analysis in South Korean Adults Stratified by Sex
Source: Nutrients. 2024 Jul 4;16(13):2136. doi: 10.3390/nu16132136 (PMC11242964; doi:10.3390/nu16132136)
Supplement: Supplementary file 1 [file nutrients-16-02136-s001.zip › Supplementary Table S2.pdf]

Supplementary Table S2. Results of subgroup analysis stratified by age (using each age group's average).

Variables	Male									Female								
	Inactive	Insufficiently active				Sufficiently active				Inactive	Insufficiently active				Sufficiently active			
		Above average Phase Angle[a]				Above average Phase Angle[a]					Above average Phase Angle[a]				Above average Phase Angle[a]			
	aOR[b]	aOR[b]	95% CI			aOR[b]	95% CI			aOR[b]	aOR[b]	95% CI			aOR[b]	95% CI		
**Age**																		
19~28	1.000	1.083	0.359	-	3.269	1.443	0.649	-	3.208	1.000	2.791	0.963	-	8.087	3.275	1.355	-	7.917
29~39	1.000	2.865	1.130	-	7.261	4.673	2.239	-	9.753	1.000	0.997	0.460	-	2.164	1.897	1.051	-	3.425
40~49	1.000	0.966	0.439	-	2.128	1.926	1.042	-	3.561	1.000	1.563	0.768	-	3.184	1.284	0.768	-	2.149
50~59	1.000	1.578	0.733	-	3.396	1.793	0.859	-	3.745	1.000	0.783	0.414	-	1.481	0.860	0.460	-	1.607
60~69	1.000	1.103	0.545	-	2.231	1.416	0.731	-	2.742	1.000	0.917	0.405	-	2.072	1.843	1.066	-	3.187
70~	1.000	1.709	0.699	-	4.182	1.208	0.586	-	2.490	1.000	0.733	0.292	-	1.840	0.619	0.210	-	1.823

Abbreviations: aOR, adjusted odds ratio; CI, confidence interval

[a]Average phase angle was calculated for each sex and age group. For males, 6.17°, 6.23°, 6.12°, 5.96°, 5.54°, 4.93°, for females, 4.90°, 5.10°, 5.02°, 5.04°, 4.85°, 4.39°, respectively.

[b]Adjusted for the amount of physical activity, age, body mass index, educational level, alcohol status, smoking status, region of residence, marital status, income level, employment status, sleep duration, presence of diabetes, high blood pressure, asthma, and kidney disease.
