# Supplementary material for: Association between Physical Activity and Phase Angle Obtained via Bioelectrical Impedance Analysis in South Korean Adults Stratified by Sex
Source: Nutrients. 2024 Jul 4;16(13):2136. doi: 10.3390/nu16132136 (PMC11242964; doi:10.3390/nu16132136)
Supplement: Supplementary file 1 [file nutrients-16-02136-s001.zip › Supplementary Table S3.pdf]

Supplementary Table S3. Association between the amount of physical activity and above-average phase angle (physical activity divided into Q1–Q4).

Variables	Male				Female			
	Above average Phase Angle[a]				Above average Phase Angle[a]			
	aOR[b]	95% CI			aOR[b]	95% CI		
**Physical activity**								
Inactive	1,000							
Q1 (>0 and ≤420)	1.856	1.185	-	2.906	1.019	0.699	-	1.487
Q2 (>420 and ≤960)	1.477	0.967	-	2.255	1.204	0.900	-	1.611
Q3 (>960 and ≤1920)	1.993	1.235	-	3.218	1.200	0.824	-	1.747
Q4 (>1920)	2.061	1.234	-	3.441	1.582	1.047	-	2.391

Abbreviations: aOR, adjusted odds ratio; CI, confidence interval

[a]Average phase angle: 5.77° for males and 4.88° for females

[b]Adjusted for the amount of physical activity, age, body mass index, educational level, alcohol status, smoking status, region of residence, marital status, income level, employment status, sleep duration, presence of diabetes, high blood pressure, asthma, and kidney disease.
