# Supplementary material for: Association between Physical Activity and Phase Angle Obtained via Bioelectrical Impedance Analysis in South Korean Adults Stratified by Sex
Source: Nutrients. 2024 Jul 4;16(13):2136. doi: 10.3390/nu16132136 (PMC11242964; doi:10.3390/nu16132136)
Supplement: Supplementary file 1 [file nutrients-16-02136-s001.zip › Supplementary Table S4.pdf]

Supplementary Table S4. Association between the amount of physical activity and the Hispanic phase angle cutoff.

Variables	Male				Female			
	Above cutoff Phase Angle[a]				Above cutoff Phase Angle[a]			
	aOR[b]	95% CI			aOR[b]	95% CI		
**Physical activity[c]**								
Inactive	1.000				1.000			
Insufficiently active	1.674	1.072	-	2.615	1.208	0.857	-	1.704
Sufficiently active	1.878	1.385	-	2.548	1.483	1.122	-	1.961

Abbreviations: aOR, adjusted odds ratio; CI, confidence interval

[a]Cutoff phase angle: 6.22° for males and 5.14° for females

[b]Adjusted for age, body mass index, educational level, alcohol status, smoking status, region of residence, marital status, income level, employment status, sleep duration, and presence of diabetes, high blood pressure, asthma, and kidney disease.

[c]Divided based on energy expenditure (multiplicity of 4.0 METs for moderate-intensity physical activity, 8.0 METs for vigorous-intensity physical activity). 'Inactive' if 0 MET-min/week, 'insufficiently active' if <600 MET-min/week, and 'sufficiently active' if >600 MET-min/week.
