# Supplementary material for: Association between Physical Activity and Phase Angle Obtained via Bioelectrical Impedance Analysis in South Korean Adults Stratified by Sex
Source: Nutrients. 2024 Jul 4;16(13):2136. doi: 10.3390/nu16132136 (PMC11242964; doi:10.3390/nu16132136)
Supplement: Supplementary file 1 [file nutrients-16-02136-s001.zip › Supplementary Table S5.pdf]

**Supplementary Table S5**. Association between specific physical activity (degree of muscle-strengthening activity) and above-average phase angle.

| Variables | Male | | | | Female | | | |
|---|---|---|---|---|---|---|---|---|
| | Above average Phase Angle[a] | | | | Above average Phase Angle[a] | | | |
| | aOR[b] | 95% CI | | | aOR[b] | 95% CI | | |
| **Physical activity[c]** | | | | | | | | |
| Inactive | 1.000 | | | | 1.000 | | - | |
| Insufficiently active with no muscle-strengthening activity[d] | 1.054 | 0.649 | - | 1.711 | 0.958 | 0.671 | - | 1.368 |
| Insufficiently active with insufficient muscle-strengthening activity[d] | 1.079 | 0.339 | - | 3.435 | 3.254 | 0.571 | - | 18.553 |
| Insufficiently active with sufficient muscle-strengthening activity[d] | 3.272 | 1.780 | - | 6.017 | 1.373 | 0.749 | - | 2.514 |
| Sufficiently active with no muscle-strengthening activity[d] | 1.610 | 1.028 | - | 2.522 | 1.125 | 0.790 | - | 1.603 |
| Sufficiently active with insufficient muscle-strengthening activity[d] | 1.827 | 0.639 | - | 5.226 | 2.675 | 0.761 | - | 9.396 |
| Sufficiently active with sufficient muscle-strengthening activity[d] | 2.365 | 1.520 | - | 3.681 | 1.662 | 1.133 | - | 2.438 |

Abbreviations: aOR, adjusted odds ratio; CI, confidence interval

[a]Average phase angle: 5.77° for males and 4.88° for females

[b]Adjusted for age, body mass index, educational level, alcohol status, smoking status, region of residence, marital status, income level, employment status, sleep duration, and presence of diabetes, high blood pressure, asthma, and kidney disease.

[c]Divided based on energy expenditure (multiplicity of 4.0 METs for moderate-intensity physical activity, 8.0 METs for vigorous-intensity physical activity). 'Inactive' if 0 MET-min/week, 'insufficiently active' if <600 MET-min/week, and 'sufficiently active' if >600 MET-min/week.

[d]'No muscle-strengthening activity': does not engage in muscle-strengthening activity, 'insufficient muscle-strengthening activity': engages in one day of muscle-strengthening activity per week, and 'sufficient muscle-strengthening activity': engages in more than one day of muscle-strengthening activity per week
