# Supplementary material for: Association between Physical Activity and Phase Angle Obtained via Bioelectrical Impedance Analysis in South Korean Adults Stratified by Sex
Source: Nutrients. 2024 Jul 4;16(13):2136. doi: 10.3390/nu16132136 (PMC11242964; doi:10.3390/nu16132136)
Supplement: Supplementary file 1 [file nutrients-16-02136-s001.zip › Supplementary Table S6.pdf]

Supplementary Table S6. Association between the amount of physical activity and above-average phase angle (with reference to below-average phase angle[a]).

Variables		Q1[a]				Q2[a]				Q3[a]				Q4[a]			
		aOR[b]	95% CI			aOR[b]	95% CI			aOR[b]	95% CI			aOR[b]	95% CI		
Male	**Physical activity[c]**																
	Inactive	1.000				1.000				1.000				1.000			
	Insufficiently active	1.133	0.745	-	1.722	1.553	0.884	-	2.730	2.147	1.203	-	3.829	1.694	0.890	-	3.226
	Sufficiently active	1.386	0.902	-	2.129	2.053	1.212	-	3.480	2.031	1.276	-	3.233	3.676	2.150	-	6.286
Female	**Physical activity[c]**																
	Inactive	1.000				1.000				1.000				1.000			
	Insufficiently active	0.930	0.608	-	1.423	1.043	0.662	-	1.641	1.560	0.919	-	2.648	0.904	0.549	-	1.490
	Sufficiently active	1.200	0.843	-	1.707	1.211	0.842	-	1.741	1.145	0.730	-	1.797	1.958	1.294	-	2.961

Abbreviations: aOR, adjusted odds ratio; CI, confidence interval

[a]Average phase angle: 5.77° for males and 4.88° for females. For males, Q1, Q2, and Q3 were 6.0°, 6.2°, and 6.6°, respectively. For females, Q1, Q2, and Q3 were 5.0°, 5.2°, and 5.4°, respectively.

[b]Adjusted for age, body mass index, educational level, alcohol status, smoking status, region of residence, marital status, income level, employment status, sleep duration, and presence of diabetes, high blood pressure, asthma, and kidney disease.

[c]Divided based on energy expenditure (multiplicity of 4.0 METs for moderate-intensity physical activity, 8.0 METs for vigorous-intensity physical activity). 'Inactive' if 0 MET-min/week, 'insufficiently active' if <600 MET-min/week, and 'sufficiently active' if >600 MET-min/week.
